# Zoom in on Antibody Aggregates: A Potential Pitfall in the Search of Rare EV Populations

**DOI:** 10.3390/biomedicines9020206

**Published:** 2021-02-18

**Authors:** Rikke W. Rasmussen, Jaco Botha, Frederik Prip, Mathilde Sanden, Morten H. Nielsen, Aase Handberg

**Affiliations:** 1 Department of Clinical Biochemistry, Aalborg University Hospital, 9000 Aalborg, Denmark; r.wehner@rn.dk (R.W.R.); j.botha@rn.dk (J.B.); frederik.prip@clin.au.dk (F.P.); mathildesanden@hotmail.com (M.S.); mohn@rn.dk (M.H.N.); 2 Department of Clinical Medicine, Aalborg University, 9000 Aalborg, Denmark

**Keywords:** high-resolution flow cytometry, extracellular vesicles, protein aggregates

## Abstract

High-resolution flow cytometers (hFCM) are used for the detection of extracellular vesicles (EV) in various biological fluids. Due to the increased sensitivity of hFCM, new artifacts with the potential of interfering with data interpretation are introduced, such as detection of antibody aggregates. The aim of this study was to investigate the extent of aggregates in labels commonly used for the characterization of EVs by hFCM. Furthermore, we aimed to compare the efficacy of centrifugation and filtering treatments to remove aggregates, as well as to quantify the effect of the treatments in reducing aggregates. For this purpose, we labeled phosphate buffered saline (PBS) with fluorescently conjugated protein labels and antibodies after submitting them to 5, 10, or 30 min centrifugation, filtering or washed filtering. We investigated samples by hFCM and quantified the amount of aggregates found in PBS labeled with untreated and pre-treated labels. We found a varying amount of aggregates in all labels investigated, and further that filtering is most efficient in removing all but the smallest aggregates. Filtering protein labels can reduce the extent of aggregates; however, how much remains depends on the specific labels and their combination. Therefore, it is still necessary to include appropriate controls in a hFCM study of EVs.

## 1. Introduction

Recently, high-resolution flow cytometers (hFCM) have become available, enabling the detection of extracellular vesicles (EV) in various biological fluids. EVs are small (50–1000 nm) membrane-encapsulated particles that are released from cells by various stimuli specific to cell type and disease pathophysiology [1]. EVs are representative of their parent cell and hence express membrane proteins that are specific to the parent cell and represent the biological state of that particular cell [1]. Furthermore, EVs are involved in intercellular communication via the transfer of DNA, RNA, proteins, and bioactive lipids [1].

Flow cytometry is a widely used method for the characterization of EV populations [2]. Although conventional flow cytometry lacks the sensitivity to capture EV populations of small sizes (<270–600 nm), hFCM, with its lower detection limit, holds great potential for phenotyping EV-populations [3]. Due to the increased sensitivity of hFCM, it also introduces new artifacts with the potential of interfering with data interpretation, such as antibody (ab) aggregates [4].

It is the nature of proteins to strive towards the most energetically favorable and native state of three-dimensional structure [5]. However, this mechanism can be disturbed if proteins are exposed to stressing conditions, which will push the equilibrium towards a more aggregation-prone state. If a protein becomes partially unfolded, exposing so-called hot spots prone for aggregation, structural rearrangements of the protein structure can occur, leading to irreversible aggregation. This step is considered to be the rate-limiting step, after which subsequent growth of the aggregate is much faster [5,6]. This mechanism is also applicable for fluorescently labeled antibodies and proteins (all designated as labels from now on), which are commonly used for EV studies by flow cytometry detection. Many different factors of the production processes during manufacturing, as well as downstream protocols and handling, may infer stress on labels before they are used for EV labeling [6]. Furthermore, they consist of a complex mixture of different types of fluorophores conjugated to different types and sequences of antibody molecules or other EV detecting proteins such as lactadherin, in various kinds of buffers, all variables that potentially affect aggregation [5,6]. Together, the chosen labels and protocol procedures may give rise to aggregates of similar size and fluorescence range as EVs, and thereby potentially interfere with EV characterization by hFCM analysis.

Previous studies report that aggregates in commercially produced fluorescence-labeled antibodies can be detected by conventional flow cytometry [4,7,8] and hFCM [9,10] and potentially confound the interpretation of data. Several potential solutions have been proposed, like centrifugation [7,11,12,13,14] or filtration of labels before use [8], the use of labeled buffer controls [10], or of detergent treated samples as controls [4]. However, there are no studies specifically designed to describe this issue.

The aim of this study was to investigate the extent of aggregates in labels commonly used for the characterization of EVs by hFCM. Furthermore, we aimed to compare the efficacy of three different centrifugation procedures, as well as two different filtering procedures to remove aggregates, as well as quantify how much the treatments could reduce potential aggregates. Finally, we tested whether labels are still functional after treatment for labeling EVs in platelet-poor plasma.

## 2. Experimental Section

### 2.1. Samples

Primary sample material: Dulbecco’s Phosphate Buffered Saline (Merck KGaA, Darmstadt, Germany, Cat. No. D8537), (PBS). PBS is used for the detection of aggregates in labels, and thereby to compare the impact of different pre-treatments of labels on the number of aggregates.

Secondary sample material: Platelet poor plasma (PPP). Blood for this study was collected from anonymous healthy Danish blood donors and kindly donated by the Danish Blood Bank (Department of Clinical Immunology, Aalborg University Hospital, Aalborg, Denmark) in accordance with local ethical regulations regarding health scientific research on anonymous human biological material (Committee law part 4, §14, No. 3) and the Helsinki Declaration. Blood was collected into BD Vacutainer™ 6NC tubes containing a final concentration of 0.0105M NA3 citrate (BD Biosciences, San Jose, Ca, USA, Cat. No. 366575) and mixed by gently inverting tubes 10 times. PPP was prepared within 1 h after blood collection as described previously [15] by two cycles of centrifugation at 2500× *g* for 15 min at room temperature. After the final centrifugation step, PPP was pooled, aliquoted, and stored at −80 °C until further use. Prior to staining, PPP was thawed at room temperature (RT), vortexed, and subjected to centrifugation at 1850× *g* for 5 min at 4 °C, and the supernatant transferred to a new tube and subsequently aliquoted to tubes pre-chilled on ice for staining.

PPP is used for gating purposes as described in Section 2.7 and for testing if filtered labels are functional.

### 2.2. Labels

The following fluorescently conjugated protein labels and antibodies were used in this study: bovine lactadherin-fluorescein isothiocyanate (FITC) (83 µg/mL; Hematologic Technologies Inc., Essex, VT, USA), mouse monoclonal anti-human CD36-phycoerythrin (PE) (200 µg/mL, Clone 5-271, BioLegend Inc., San Diego, CA, USA), mouse monoclonal anti-human CD62E-allophycocyanin (APC) (100 µg/mL, Clone HAE-1f, BioLegend Inc., San Diego, CA, USA), mouse monoclonal anti-human Leukotriene B4 R1-Alexa Fluor700 (AF700) (700 µg/mL, Clone 202/7B1, Novus Biologicals, Abingdon, UK), mouse monoclonal anti-human CD14-APC (200 µg/mL, Clone 63D3, BioLegend Inc., San Diego, CA, USA), mouse monoclonal anti-human CD9-PE (20 µg/mL, Clone HI9a, BioLegend Inc., San Diego, CA, USA), mouse monoclonal anti-human CD41a-Brilliant Violet510 (BV510) (50 µg/mL, Clone HIP8, BD, San Jose, CA, USA), mouse monoclonal IgG2a, κ-PE (200 µg/mL, MOPC-173, BioLegend Inc., San Diego, CA, USA), mouse monoclonal IgG1, κ-APC (200 µg/mL, Clone MOPC-21, BioLegend Inc., San Diego, CA, USA), mouse monoclonal IgG2a-AF700 (50 µg/mL, Clone 20102, Novus Biologicals, Abingdon, UK) mouse monoclonal IgG1, κ-PE (200 µg/mL, Clone MOPC-21, BioLegend Inc., San Diego, CA, USA), mouse monoclonal IgG1, κ-BV510 (200 µg/mL, Clone X40, BD, San Jose, CA, USA). See Table 1 for details.

### 2.3. Label Panels

Label panels of specific ab and their isotype controls were prepared at the start of each analysis day (details are given in Table 1). Panel 1 (P1) and panel 2 (P2) were used to study pre-treatment of labels in PBS, and panel 3 (P3) was used for testing results on an independent ab panel on PPP.

### 2.4. Pre-Treatment of Labels

The extent of aggregates in untreated (U) labels was studied by pipetting directly from the freshly vortexed vial of label.

Additionally, we tested five different pre-treatments of P1 and P2 in order to determine the efficacy of three high-speed centrifugation protocols of labels, as well as the filtration of labels prior to staining. Prior to applying each pre-treatment protocol, all label vials were briefly vortexed in order to homogenize the labels as with the untreated labels. For the high-speed centrifugation protocols, labels were subjected to centrifugation at 17,000× *g* for either 5 (C5), 10 (C10), or 30 (C30) minutes at 4 °C, and care was taken to prevent stirring in the subsequent procedures, after which panels were prepared from the top-most supernatant. For labels subjected to filtration (F), labels were pipetted directly on top of 0.45 µm hydrophilic PVDF centrifugal filters (Merck KGaA, Darmstadt, Germany, Cat. No. UFC30HVNB) and centrifuged at 12,000× *g* for 4 min according to the manufacturer’s recommendation, and the filtrate was used for labeling samples. In addition, the effect of pre-washing the filter (WF) was investigated by adding 500 µL PBS on top of the PVDF filter and centrifuging as above. Residual PBS was wiped off the bottom of the filter before it was transferred to a new centrifuge tube. Labels were then filtered by the same procedure as described for F.

### 2.5. Staining of Samples

PBS or PPP was incubated with either specific or isotype control label of P1, P2, or P3, as specified in Table 1. Labels were either pipetted directly from ab-vial (C and U treatments) or from a master mix of all labels (F and WF treatments). Samples were incubated in the dark for 30 min on ice and then diluted 17-fold with PBS. Diluted samples were kept on ice in the dark until analysis. In addition, a detergent lysis control was prepared for each PPP sample stained with either of the specific label-master mixes by incubation with Triton X-100 (final concentration: 1% *v*/*v*) for at least 30 min on ice.

### 2.6. Setup

**Figure 1:**
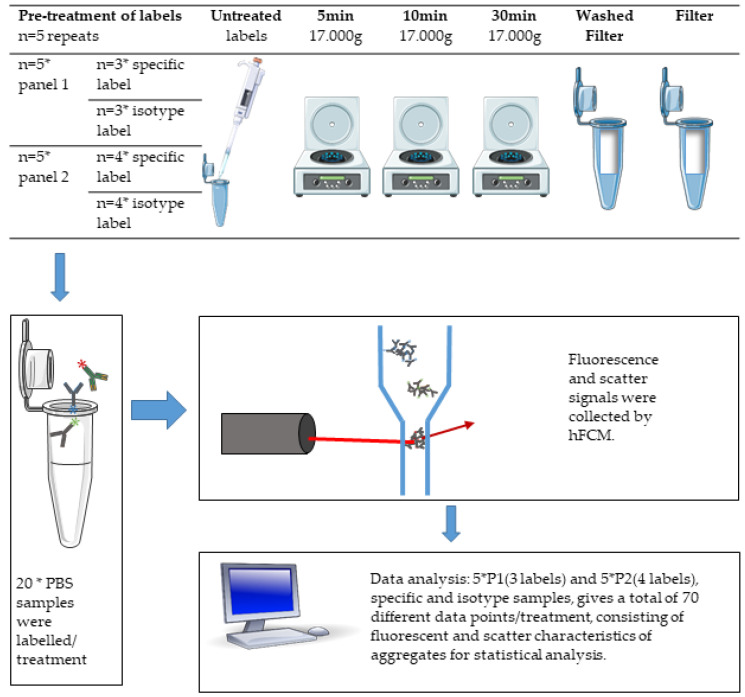
Flowchart of pre-treatment, labeling, high-resolution flow cytometer (hFCM) analysis, and data analysis.

In order to investigate and account for pre-analytical variability in the process of preparing label panels and measuring samples, all conditions were measured in five independent replicates in PBS. Labels were prepared separately for each replicate and pre-treatment, and replicates were analyzed on separate days. For an overview, see Figure 1.

In addition, single replicates of PPP labeled with either P1 or P2 were measured and used as gating controls for subsequent analyses of labeled PBS samples. In order to test the functionality of master mixes subjected to filtration, PPP was stained with P3 or its matched isotype controls (Table 1).

### 2.7. Analysis of Label-Aggregates by hFCM

Samples were analyzed on an Apogee A60 Micro-PLUS high-resolution flow cytometer (Apogee Flow Systems, Hertfordshire, UK) equipped with a 200 mW 488 nm diode laser set to 100 mW, a 180 mW 638 nm diode laser set to 100 mW, and a 300 mW 405 nm diode laser set to 190 mW. Light scatter signals were collected off of the 405 nm laser into highly sensitive photon electron multiplier tubes (PMTs) after being separated from fluorescence signals by an LP415 long pass filter. Directions and PMT settings are indicated in Table 2. Fluorescence signals were collected off of lasers into PMTs fitted with bandpass filters, also indicated in Table 2.

Samples were acquired for 120 s at a constant sample flow rate of 0.75 µL/min and sheath pressure of 150 mBar. In order to exclude excessive background noise, a medium angle light scatter triggering threshold was set above background, acquiring <100 events per second in unstained PBS and kept at the same level throughout the experiment. Both untreated and pre-treated labels were tested in PBS in order to collect data exclusively arising from aggregates. For control of purity of PBS and the fluidics system in the Flow Cytometer, one pure PBS-sample was analyzed by the same protocol as the labeled samples each day. One labeled and one matched isotype control sample was prepared of PPP for each panel for setting appropriate gates to define label-positive populations. Gating was conducted as shown in Figures S1 and S2 using FlowJo version 10.5.3 (FlowJo LLC, BD Biosciences, San Jose, CA, USA). First, events at time points with erratic event rates were removed from files, as these were considered not to be representative of the actual concentration of label-positive events due to changes in the sample core width and increased background fluorescence signals. Second, EV-size gates were established below the first percentile of 1300 nm silica-beads (ApogeeMix Calibration Beads, Apogee Flow Systems, Hertfordshire, UK) in a scatterplot of small-angle light scatter versus large-angle light scatter and transferred to all files analyzed in the same analysis session (Figure S1). Third, a fluorescence gate was established in the FITC-channel on the ninety-ninth percentile of triton X-100 treated PPP stained with specific label master mixes in order to define lactadherin-positive events (Figure S2). In a similar manner, fluorescence gates were established on the ninety-ninth percentiles of PE, APC, AF700, and BV510 channels on PPP stained with isotype control master mixes (Figure S2). These gates were transferred to all stained PBS samples as appropriate with regards to label-panel. In this way, we could assure that the calculated concentrations for each label/fluorophore are in the appropriate size and fluorescence range for EV studies.

### 2.8. Statistical Data Analysis

All data processing and statistical data analysis was conducted in R v. 3.5.1 (R Core Team, Vienna, Austria) in RStudio v. 1.1.456. Data on concentrations of label-positive particles was loaded from .xls files exported from FlowJo with the xlsx package [16], after which data for both label panels were concatenated and converted to long data using the reshape2 [17] package. Next, concentrations of label-positive events were normalized to the mean of the corresponding untreated labels and compared between treatments for each label/fluorochrome combination. Data were aggregated by panel, and all data were aggregated using pairwise Wilcoxon Rank Sum Tests from the base and stats packages native to R without adjusting *p*-values for multiple comparisons. Statistical significance was set to *p* < 0.05. Finally, figures of summarised concentration data were plotted using the ggplot2 [18] and grid [19] packages.

## 3. Results

### 3.1. Aggregates Were Present in Untreated Labels

In order to investigate the presence of fluorescent aggregates in untreated labels, we tested five different specific labels and their matched isotype controls combined in two different panels (P1 or P2) in PBS. By using the EV gates established in correspondingly labeled PPP, fluorescent particles were detected in labeled PBS. We compared the mean of positive event concentrations detected in unlabelled PBS in each channel to the mean of positive event concentration in PBS with untreated labels.

**Figure 2:**
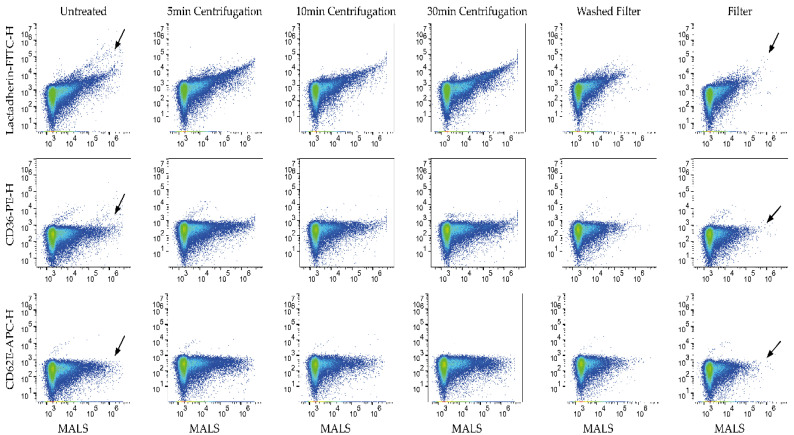
Representative scatterplots of phosphate buffered saline (PBS) labeled with P1 specific label. Medium angle light scatter (MALS) versus fluorescence. Colum’s represent untreated, centrifugated, and filtered labels.

When looking at compensated and gated flow cytometry data, it was evident that label aggregates were present in the fluorescence-positive gates in stained PBS samples (untreated labels P1 Figure 2 and P2 Figure S3). For comparison, see Figure S4 showing PBS only). The addition of labels to PBS resulted in increased concentrations of fluorescence-positive (aggregated labels) events for all labels investigated (Table 3).

In addition, some variability was observed for labels with the same fluorochrome between panels and also between specific antibodies and their isotype controls.

Interestingly, large variations in the mean concentration of the lactadherin-FITC could be observed across different panels and also between the specific antibody panel and its isotype control panel. This finding was further supported by observations for isotype-APC, CD36-PE, and isotype-PE, which all varied in a similar fashion across panels (Table 3). When comparing labels with different fluorophores, concentrations of aggregated labels varied somewhat and even more so when comparing their fold difference from unlabelled PBS ranging from 5.6 to 648-fold (Table 3).

Thus, label aggregates were present in all antibody/label and fluorophore combinations, and their extent varied depending on the panel they were in, which could complicate data interpretation to varying degrees.

### 3.2. Filtration of Labels Is the Most Efficient of the Tested Methods in Removing Label Aggregates

In order to investigate the general effects of pre-treatments regardless of label, we normalized all data to mean U concentrations and compared each pre-treatment method to all other conditions.

**Figure 3:**
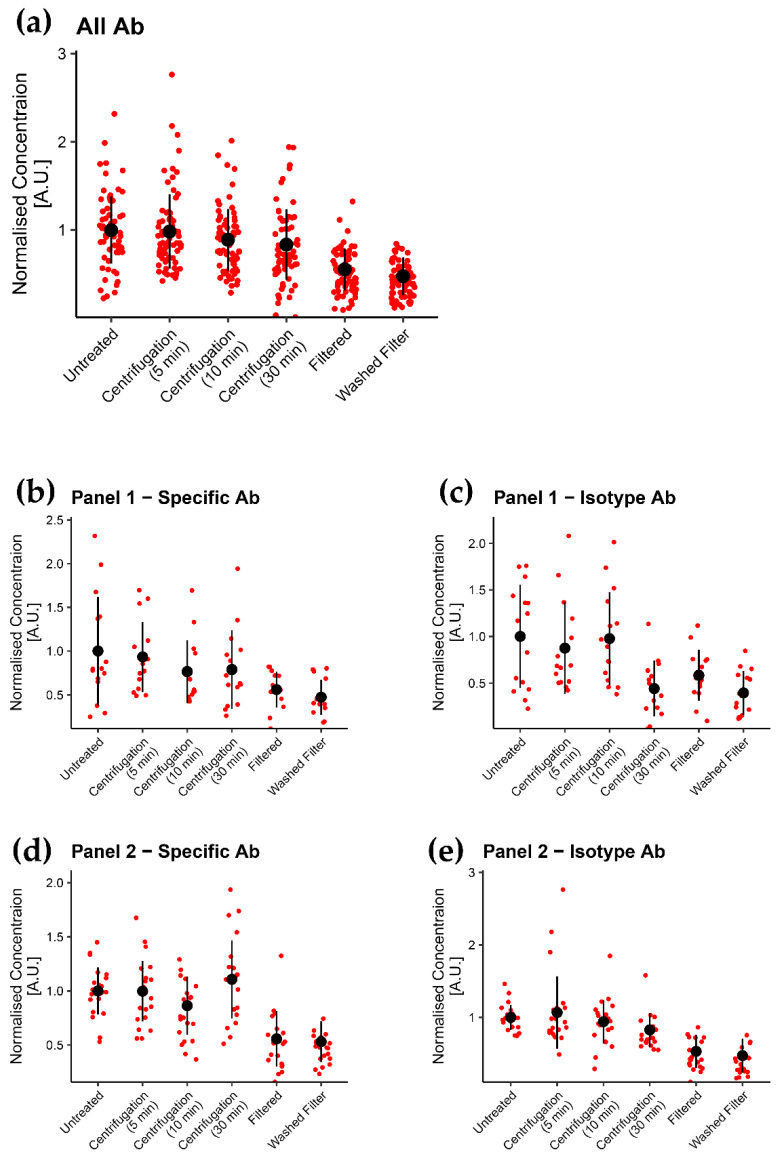
(**a**): Distribution of concentrations of all labels for each treatment when normalized to the average of all untreated labels. (**b**–**e**): distribution of concentrations of all labels for P1-specific, P1-isotype, P2-specific, and P2-isotype label, respectively, for each treatment when normalized to the average of all untreated labels.

When aggregating all data across all panels and their isotype controls (*n* = 70 per condition), we demonstrated that both filtered (F) and washed filter (WF) methods significantly reduced aggregates compared to the untreated (U) (*p* < 0.001) method and were significantly more effective in removing aggregates compared to any of the centrifugation (C5, C10, and C30) pre-treatment methods (*p* < 0.001, Figure 3, All Ab and Table S1, All Panels/All Labels). Of note, among the centrifugation pre-treatment procedures, only 30 min C significantly reduced aggregates compared to U (*p* < 0.05). Furthermore, less variation is introduced by F and WF treatment (Figure 3 and Table 4).

By looking at data for all fluorophores in the same panel aggregated together (*n* = 15 for P1, *n* = 20 for P2), only F and WF consistently reduced aggregates in the four panels studied compared to U when including all labels (*p* < 0.05, Figure 2 and Figure 3b–e, Figure S3 and Tables S1 and S2). Centrifugation procedures only induced a significant reduction of aggregates compared to U by C30 on isotype panels (*p* < 0.05). Furthermore, F and WF were significantly more efficient compared to centrifugation procedures when applied on P2 (*p* < 0.001); however, less consistent on P1 (*p* = 0.002–0.547) (Tables S1 and S2).

Looking at the individual label- fluorochrome combinations (*n* = 5 per combination), only F and WF reduce aggregates of all labels in P2, both specific and isotype label, compared to U labels (*p* = 0.008–0.032), with the exception of CD14-APC, which did not differ significantly to untreated in F (Figures S7 and S8 and Table S1). However, both F and WF pre-treatment were less consistent in reducing aggregates in P1 specific and isotype label compared to U labels and only induced significant reduction in P1 CD36-PE label (*p* = 0.016–0.032, Figures S5, S6 and Table S1). For the centrifugation treatments, a significant reduction of aggregates was only seen for C30 in P2 isotype-APC and PE label and P1 isotype-APC label (*p* < 0.05). Thus, there was no consistent effect of centrifugation when applied on individual panels or fluorochromes; however, this could also arise from the small sample size in this data set (*n* = 5 per group).

### 3.3. Aggregates Were Considerably Reduced by Using Filter

Mean total event concentrations of aggregated labels were reduced 47% ± 24% by F, and 57% ± 18% by WF pre-treatment compared to concentrations of U samples (Table 4). However, no consistent aggregate reduction was observed for centrifugation procedures (2% ± 45%, 13% ± 37%, and 19% ± 42% for C5, C10, and C30, respectively). When looking at specific fluorophores, FITC label aggregates were reduced to a similar extent by F (63% ± 22%) and WF (62% ± 20%); however, WF reduced aggregates in PE, APC, and AF700 slightly better than F (PE: 49% ± 16% vs. 59% ± 18%; APC: 33% ± 26% vs. 57% ± 17%; AF700: 40% ± 16% vs. 43% ± 13%, respectively).

When looking more closely at how F and WF effect labels, it was evident that aggregates that tended to scatter more light (i.e., due to a larger size) and exhibited more fluorescence were removed by filtration (Figure 2 and Figure S3 black arrows). As such, particles scattering less light and exhibiting less fluorescence were unaffected to a large degree by F and WF, which could explain some of the variability observed for these methods. Scatterplots of centrifugation methods resemble scatterplots showing untreated labels (Figure 2 and Figure S3).

### 3.4. Filtrated Labels Are Functional; However, Some Aggregates Pass through the Filter

**Figure 4:**
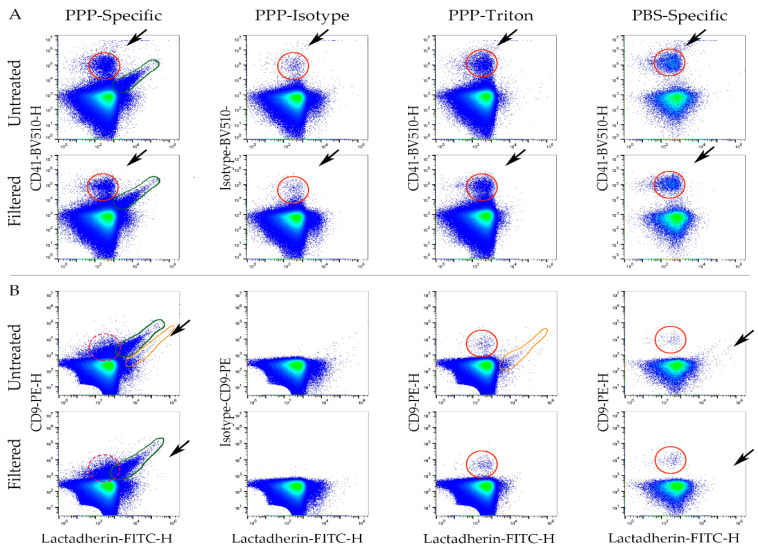
Scatterplots showing (**A**) lactadherin-FITC/CD41-BV510 or (**B**) lactadherin-FITC/CD9-PE of platelet poor plasma (PPP) labeled with panel 3 untreated or filtered labels. Corresponding isotype, triton, and labeled PBS controls are shown. Green circle: lactadherin-FITC+/CD41-BV510+ or CD9-PE+ EV− population. Red circle: CD41− or isotype-BV510+ or CD9-PE+ aggregates. Punctuated red circle: CD9-PE aggregates might be hidden among the CD9+ EV population. Orange circles: lactadherin-FITC+/CD9-PE+ population. This population is not seen in isotype or PBS control or in filtered-specific labeled PPP.

In order to test whether labels are functional after filtration and the influence in staining of EV-populations, we stained PPP with P3 and compared filtered with untreated labels and their corresponding controls as described above. The resulting scatterplots are shown in Figure 4.

Distinct populations positive for both lactadherin-FITC and either CD41-BV510 or CD9-PE could be seen in specifically labeled samples and not in isotype or detergent lysis controls, thereby suggesting the presence of EVs positive for these markers in both F and U samples (Figure 4A,B, solid green circles). In addition, label aggregates could be observed for all labels used in this panel; however, this was most clear for antibodies conjugated to BV510, which gave rise to a BV510+ population (Figure 4A, solid red circle) present in all samples and controls. A population of events with slightly higher scatter and fluorescence values (Figure 4A,B, black arrows) was; however, largely removed in samples stained with filtered labels. In the lactadherin-FITC/CD9-PE scatterplots, we found CD9-PE aggregates in the triton, and PBS controls as well (Figure 4B, solid red circles), again both for untreated and filtered samples. It is not possible to distinguish if this population of aggregates were present in the PPP-specific samples, as these events fell in the same region on this scatter plot as the CD9+ EV population (dotted red circles). One FITC+/PE+ population was found in the U specific labeled PPP, and more faint was seen in the triton control. However, this population was not seen in the F specific labeled PPP (orange circle) (Figure 4).

## 4. Discussion

In order to reduce sources of error when using small-particle flow cytometry for EV studies, it is an important part of standardization to apply staining procedures inducing as little possible bias and background noise arising from label aggregates across protocols and workflows from the beginning to the end of the study. In this study, we have focused on creating a more informed basis for deciding how to pre-treat labels in order to minimize positive event counts due to fluorescent label aggregates in samples.

### 4.1. Aggregates in the Size Range of EVs Are Present in Labelled PBS

As expected, and in line with previous studies showing events in buffer containing fluorescently labeled antibodies [7,10,20], we found aggregates in PBS labeled with U labels compared to unlabelled PBS. The increase in concentrations of all fluorophores was significant in all panels.

We designed two label panels partly in order to investigate several different labels and partly to see if the same label would behave differently when in combination with other antibodies in different panels. Expectedly, we found large variations in concentrations between different labels in the same panel. We have previously experienced large differences in aggregates between different types of labels (i.e., antibodies against different markers), labels with different fluorophores conjugated to them (i.e., FITC, PE, etc.), but also between different lot numbers of the same label (data not shown). Similar observations have been made by Görgens et al. comparing labels from different manufactures [10]. Previously, H.C. Inglis et al. [20] has studied the effect of three different filters and one centrifugation protocol on aggregates in labels. However, in this study, we present a more extensive setup and, furthermore, a detailed data analysis incorporating statistical assessment of the different treatments compared to each other, applied to two different panels and their respective isotype controls. Interestingly we also found variation among the same label in this study. Lactadherin is a well-known molecule used to label phosphatidylserine (PS) on the surface of cells and EVs, and the presence of PS has previously been used in PPP samples to define EVs and exclude non-EV-particles [21,22]. A comparable tendency was observed for lactadherin-FITC and isotype-APC. Both results could, to some extent, be accounted for by differing lot numbers, which might have a variable amount of aggregates present due to small variations in the manufacturing process that can potentially influence the aggregation of proteins [6]. Furthermore, the context and downstream protocol may also introduce aggregates as demonstrated by consistently higher concentrations of aggregates in P2 vs. P1 of CD36− and isotype-PE (same lot numbers) and in P2 isotype vs. P2 specific of lactadherin (see Table 3 and Table S5 for concentrations of individual labels in each panel, for untreated and for each treatment, respectively). These relative differences are independent of pre-treatment, which suggests that the combination of antibodies in a panel could also potentially affect the concentration of label aggregates in a specific sample. This highlights the importance of testing labels in the buffer, ideally both as a master mix of all labels and each label alone.

### 4.2. Efficacy of Treatments

In this study, we expected to see differences in the efficacy of different label treatments to remove label aggregates and that these differences are consistent regardless of label or panel consisting of several different labels. The F and WF method proved to be the most robust in terms of removing most aggregates, being the most reliable method in terms of reproducibility across days, and having the least variability compared to C and U methods.

This finding is in line with the results of the study by Inglis et al. comparing total event count in samples containing either untreated labels or labels subjected to centrifugation or filtering [20]. They too found filtering to be more effective than 5 min centrifugation. Additionally, they compared filters with three different pore sizes and found them to be equally effective. In contrary to our results, Aass et al. [7] found centrifugation to be sufficient to remove aggregates. However, in both studies, a less sensitive flow cytometer was used, and therefore smaller aggregates may still have been present, which would have remained under the detection threshold of their cytometer after centrifugation. There could be several possible explanations for the discrepancies between our results for centrifugation treatments and those of Inglis and Aass. The C method is particularly sensitive to operator errors and discrepancies in procedures between analysis days. First, it is difficult to avoid movements of the vial when pipetting after centrifugation, which could cause stirring of the aggregates into the liquid. Second, differing amounts of aggregates could also be re-absorbed into the liquid by diffusion if the amount of time between centrifugation and preparation of label panels varies between analysis days. Third, as labels are used, the volume of liquid in the vial decreases while the concentration of aggregates steadily increases, making it perpetually more difficult to avoid mixing aggregates into the liquid and transferring these to samples.

Filtering labels, on the other hand, proved to be more robust and less subject to bias throughout the entire work process. We were, however, concerned that the filter could contain impurities from production that could show up in our data, so we included the WF treatment. There was a statistically significant difference in the concentration of aggregates between F and WF methods. However, the magnitude of this difference has little practical significance.

Even though F or WF proved to be the best options of the methods tested in this study to remove aggregates from fluorescent labels, both methods had some drawbacks. While staining of PPP with filtered labels showed that labels are still functionally capable of labeling EVs, different labels and their aggregates had varying abilities to cross through the membrane. Of note, aggregates from filtered BV-510 conjugated labels show up as a distinct population in data. A reason for this could be that the fluorophores tested are of different sizes and shapes as well as surface-charge distributions might be the reason for the different efficacy of the filter towards removing the aggregates. Brilliant Violet fluorophores consist of long organic polymers of aromatic units and side-chain modifications. Aggregates of these might be able to unfold to even longer polymers capable of passing through the filter during centrifugation.

This study reveals the extent and variability of aggregates in labels and how pre-treatment affects them. Additionally, this study further highlights the importance of using adequate detergent lysis and stained buffer controls in characterization of EVs in biological samples, as populations of aggregates might be mistaken for EV-populations and confound on results. Finally, it was beyond the scope of this study to say with which magnitude this issue affects actual EV-event count and interpretation of data.

### 4.3. Reasons for Aggregation and Implications in EV Studies

Overall, results of aggregate concentrations are inconsistent between labels, fluorophores, and panel compositions. However, the variating concentrations cannot be explained fully by operator errors or different lot numbers. As labels of the same lot number display concentration levels independent of pre-treatment but apparently dependent on panel combination, other explanations must be sought. As mentioned above, the native three-dimensional structure of proteins is maintained by a delicate mechanism and equilibrium. The native structure of proteins is held in place by hydrogen bonds, salt bridges, disulfide bonds, steric hindrance, bond torsions, and hydrophobic interactions [23]. The three-dimensional folded structure can display partially unfolded patches, exposing hydrophobic patches that in turn can bind to other unfolded proteins, thereby creating a dimer. This step is still a reversible reaction. The key step is the nucleation step, where structural rearrangements occur in the protein strand. It can be α-helixes becoming β-sheet, or alteration of surface charge. This step is irreversible, and creates soluble oligomers [5,6], also called an aggregation nucleus. Further aggregation is dependent on this formation of a nucleus. A series of stressing factors can disturb the equilibrium, tipping it towards the more aggregation prone states. These factors can be temperature, pressure, freeze-thaw cycles, shaking/shearing, solvent properties as pH, ionic strength, protein concentration, and hydrophobic surface area in the solvent [6].

**Figure 5:**
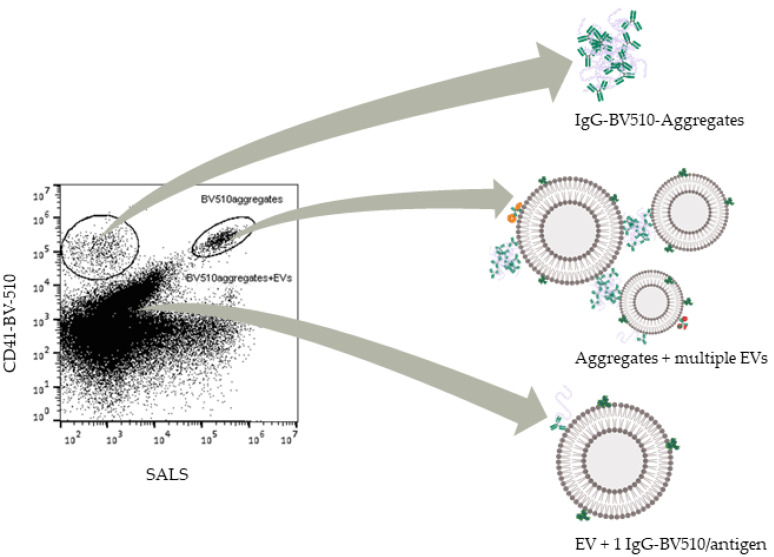
Illustration of the hypothesis of aggregates containing multiple labels and extracellular vesicles (EVs). Scatter plot of PPP labeled with lactadherin-FITC, CD41-BV510, CD36-PE, and ApoB-APC. Schematic representation of the different labels is shown in Table S3.

In accordance with the above-explained mechanism, an explanation for the differences in aggregate concentrations between P1 and P2 could be the presence of one label containing aggregation-nucleus, promoting the formation of aggregates containing multiple different labels in the master mix, which could be able to bind EVs and be a potential confounder in the analysis of EV data. Even though antibodies and labels are partially denatured and aggregate, it is still entirely possible that they are functional. In the case of antibodies such as mouse monoclonal IgG1 or IgG2 commonly used in flow cytometry characterization studies, the Fab ends of the antibodies could still be functional, while the Fc region is denatured. As such, complexes of multiple antibodies could be capable of binding multiple EVs, potentially giving rise to artificial phenotypes positive for several protein markers in a similar fashion to swarm detection [24]. To illustrate this point, a scatterplot of PPP labeled with lactadherin-FITC, CD41-BV510, CD36-PE, and ApoB-APC from another study in our lab (ongoing) is shown in Figure 5.

Here, we illustrate our hypothesis on what comprises the different populations seen in the sample after labeling. From Figure 5, a population consisting of single EVs stained with non-aggregated labels (i.e., EV + 1 IgG-BV510) can be seen close to the background, steadily increasing in both scatter and fluorescence with scatter signal correlating somewhat well with fluorescence. Above this, aggregates of IgG-BV510 can be seen with a low scatter signal and high apparent fluorescence with no apparent correlation between the signals. Finally, aggregates staining multiple EVs are seen as a clearly discriminable extension of the EV population, albeit with both higher scatter and fluorescence intensities than the EV population. Populations corresponding to aggregates staining multiple EVs can be seen in multiple fluorescent channels for labeled PPP, but not in isotype labeled PPP or detergent treated PPP (see Figure S9).

The process of aggregation is very complicated and difficult to control. This can explain the variable quality of different lot numbers of fluorescently labeled antibodies and protein-based labels when receiving them from the manufacturer. After obtaining the label, the operator should carefully consider the handling procedure. We have listed a set of points to consider in Table S5, when designing a new protocol and label panel for an EV-study. One of the factors that can influence aggregation formation its solvent properties. This might be a weakness of this study, as PBS is fundamentally different from biological samples in terms of protein concentration and hydrophobic surface area in a solvent, just to mention the most obvious. Whether this leads to more aggregates in labeled PBS compared to PPP is beyond the scope of this study. However, many EV studies are based on samples containing purified EVs that are diluted in PBS [10,20,25]. In these cases, the physical characteristics of the sample would be comparable to labeled PBS in this study. Another weakness of this study is that we have only five different observations per fluorophore per treatment. This, together with the large variation per run, makes it impossible to distinguish differences among the treatments at a single-label level. A strength of this approach, on the other hand, is that we had 10 different label-fluorophore combinations combined in four different ways, yielding a total of 70 different datapoints per treatment. This gives a good impression of the overall effect of different treatments in different panels of labels.

Overall, this study shows the importance of thorough investigation and interpretation of controls, as aggregates are ever present in labels and panels and are thus added to samples, where they can potentially confound on results. This likely applies to any kind of biological material and purification method chosen for EV analysis, although the extent to which other biological materials and methods are affected by the presence of label aggregates warrants further investigation. The results of this study further highlight the need for rigor in including controls such as the stained buffer and detergent lysis controls in order to confirm the presence of EVs, and to demonstrate and control for the extent of potential artifacts.

## 5. Conclusions

We set out to investigate the extent of aggregates in labels and which pre-treatment would be best for the reduction of aggregates in labels used in hFCM of EVs. However, the results did raise several other questions regarding the design of the labeling protocol and label panel.

Overall, the F or WF methods proved to be the best options of the ones tested here for removing aggregates before labeling samples for hFCM analysis of EVs. However, one should bear in mind that it can be difficult to remove a satisfactory amount of aggregates in labels containing high amounts of aggregates. In future studies, filters of different hydrophobicities and pore sizes should be investigated. Additionally, more research should be done on the storage of labels and labeling protocol in order to minimize the risk of inducing aggregation in the labels. Points for optimization could be incubation temperature and time, vortexing vs. gentle mixing by pipetting, surface characteristics in tubes and pipette tips, sequential labeling versus master mix, and choice of antibodies and fluorophores. Nonetheless, it might not be realistic to find a “one size fits all” protocol for labeling EV samples for hFCM in order to avoid aggregates interfering with results. Finally, one must bring into consideration which population of EVs is being searched for, and whether aggregates (if present after appropriate pre-treatment) interfere with EV population count, and if so, whether it is possible to correct during data acquisition and processing. This reasoning might be applicable for other methods used for EV studies as well as, e.g., nanoparticle tracking analysis or super-resolution microscopy. Future studies in label aggregates could benefit from including some of these additional techniques.

## Figures and Tables

**Table 1:** Labels used in this study.

Panel	Specific Label	Isotype Control Label
1	5 µL bovine lactadherin-FITC, Cat. #BLAC-FITC, lot #GG0420	5 µL bovine lactadherin-FITC, Cat. #BLAC-FITC, lot #GG0420
	2.5 µL mouse monoclonal anti-humanCD36-PE, Cat. #336206, lot #B219025	2.5 µL mouse monoclonal IgG2a, κ-PE, Cat. #400214, lot #B213581
	2.5 µL mouse monoclonal anti-human CD62E-APC, Cat. #336012, lot #B194637	2.5 µL mouse monoclonal IgG1, κ-APC, Cat. #400122, lot #B210432 ^1^
2	5 µL bovine lactadherin-FITC, Cat. #BLAC-FITC, lot #HH0216	5 µL bovine lactadherin-FITC, Cat. #BLAC-FITC, lot #HH0216
	2.5 µL mouse monoclonal anti-human CD36-PE, Cat. #336206, lot #B219025	2.5 µL mouse monoclonal IgG2a, κ-PE, Cat. #400214, lot #B213581
	2.5 µL mouse monoclonal anti-human CD14-APC, Cat. #367118, lot #B230117	2.5 µL mouse monoclonal IgG1, κ-APC, Cat. #400122, lot #B216781
	2.5 µL mouse monoclonal anti-human Leukotrine B4 R1-AF700, Cat. #NB100-64831AF700, lot #0709-052517 ^2^	2.5 µL mouse monoclonal IgG2a-AF700, Cat. #IC003N, lot #ACIT025061
3	5 µL bovine lactadherin-FITC, Cat. #BLAC-FITC, lot #HH0216	5 µL bovine lactadherin-FITC, Cat. #BLAC-FITC, lot #HH0216
	2.5 µL mouse monoclonal anti-human CD41a-BV510, Cat. #563250, lot #7164967	2.5 µL mouse monoclonal IgG1, κ-BV510, Cat. #562946, lot #7282966 ^3^
	5 µL mouse monoclonal anti-human CD9-PE, Cat. #312106, lot #B251912	5 µL mouse monoclonal IgG1, κ-PE, Cat. #400112, lot #B245983 ^4^

Dilution factor (Df): ^1^ Df = 2. ^2^ Df = 14. ^3^ Df = 4. ^4^ Df = 10.

**Table 2:** Settings for Apogee A60 Micro-PLUS high-resolution flow cytometer.

Laser	Collected Signal	PMT ^1^	LP/BP ^6^ Filter
405 nm (190 mW)	SALS ^2^	545	LP415
-	MALS ^3^	405	-
-	LALS ^4^	410	-
-	BV510 ^5^	500	BP 525/40
488 nm (100 mW)	FITC ^5^	480	BP 530/40
-	PE ^5^	407	BP 575/30
638 nm (100 mW)	APC ^5^	450	BP 680/35
-	AF700 ^5^	520	LP 740

^1^ Photon multiplier tube. ^2^ Small angle light scatter. ^3^ Medium angle light scatter. ^4^ Large angle light scatter. ^5^ Fluorophores used in this study. ^6^ LP: long pass. BP: band pass.

**Table 3:** Concentration of fluorophores in PBS labeled with untreated panels, compared to unlabelled PBS.

Panel	Label	Fluorophore	Mean ^3^ Conc. ± SD (Events/µL)	FC ± SD ^4^	*p*-Value
PBS	Unlabelled	FITC (*n* = 13)	3798 ± 2360	-	-
-	-	PE (*n* = 13)	92 ± 103	-	-
-	-	APC (*n* = 13)	35 ± 64	-	-
-	-	AF700 (*n* = 13)	6642 ± 1898	-	-
P1(U) ^1^	Specific	FITC (*n* = 5)	66,234 ± 52,782	17.4 ± 13.9	0.0021
-	-	PE (*n* = 5)	10,141 ± 3558	110.1 ± 38.6	0.0018
-	-	APC (*n* = 5)	1844 ± 1401	52.0 ± 39.5	0.26
P1(U) ^1^	Isotype	FITC (*n* = 5)	69,816 ± 44,351	18.4 ± 11.7	0.0021
-	-	PE (*n* = 5)	10,069 ± 5897	109.3 ± 64	0.0087
-	-	APC (*n* = 5)	1736 ± 990	48.9 ± 27.9	0.31
P2(U) ^2^	Specific	FITC (*n* = 5)	38,179 ± 5098	10.1 ± 1.3	0.0021
-	-	PE (*n* = 5)	37,458 ± 10,780	406.6 ± 117.0	0.0005
-	-	APC (*n* = 5)	24,647 ± 9529	694.9 ± 268.7	0.0022
-	-	AF700 (*n* = 5)	25,931 ± 3521	3.9 ± 0.5	0.0018
P2(U) ^2^	Isotype	FITC (*n* = 5)	106,293 ± 21,650	28.0 ± 5.7	0.0021
-	-	PE (*n* = 5)	67,109 ± 9426	728.4 ± 102.3	0.0005
-	-	APC (*n* = 5)	63,642 ± 18,649	1794.3 ± 525.8	0.0022
-	-	AF700 (*n* = 5)	48907 ± 6233	7.4 ± 0.9	0.0018
Mean U total	N.A.	FITC (*n* = 20)	70,130 ± 41,492	18.4 ± 10.9	<0.001
-	-	PE (*n* = 20)	31,194 ± 25,239	338.6 ± 273.9	<0.001
-	-	APC (*n* = 10)	22,967 ± 27,660	648 ± 780	0.0021
-	-	AF700 (*n* = 10)	37,419 ± 13,016	5.6 ± 2.0	<0.001

^1^ Panel 1, untreated. ^2^ Panel 2, untreated. ^3^ Mean of concentration (event/µL) of positive events in pure PBS, or PBS labeled with untreated labels were calculated for all labels. ^4^ Foldchange (FC) of each fluorophore was calculated as Mean U (P1 or P2, Specific or Isotype)/Mean PBS unlabelled; *p*-value denotes label vs. unlabelled PBS.

**Table 4:** Reduction (%) of mean event concentrations (evt/µL) of fluorophores in PBS labeled with panels treated with either 5, 10, or 30 min centrifugation (C5, C10, or C30) or filtered (F) or washed filter (WF), compared to evt/µL in PBS labeled with untreated labels (Table 3).

Treatment	Fluorophore (All Panels)	Mean Conc. ± SD (Events/µL)	Reduction % (Compared to U) ± SD	CV % (Reduction)
C5	FITC (*n* = 20)	98,485 ± 72,816	−31 ± 61	-
-	PE (*n* = 20)	29,665 ± 24,401	8 ± 27	-
-	APC (*n* = 20)	19,178 ± 22,428	25 ± 30	-
-	AF700 (*n* = 10)	34,983 ± 12,557	6 ± 16	-
C5 total	All (*n* = 70)	47,091 ± 53,701	2 ± 45	2477
C10	FITC (*n* = 20)	78,410 ± 46,615	−9 ± 47	-
-	PE (*n* = 20)	26,480 ± 25,301	16 ± 31	-
-	APC (*n* = 20)	18,287 ± 23,235	33 ± 25	-
-	AF700 (*n* = 10)	32,627 ± 12,169	12 ± 18	-
C10 total	All (*n* = 70)	39,845 ± 39,594	13 ± 37	281
C30	FITC (*n* = 20)	65,824 ± 37,851	5 ± 42	-
-	PE (*n* = 20)	24,564 ± 21,156	30 ± 30	-
-	APC (*n* = 20)	19,953 ± 21,083	29 ± 53	-
-	AF700 (*n* = 10)	33,404 ± 9282	6 ± 25	-
C30 total	All (*n* = 70)	36,298 ± 31,979	19 ± 42	216
F	FITC (*n* = 20)	25,340 ± 16,192	63 ± 22	-
-	PE (*n* = 20)	15,281 ± 12,283	49 ± 16	-
-	APC (*n* = 20)	13,875 ± 16,425	33 ± 26	-
-	AF700 (*n* = 10)	22,630 ± 10,008	40 ± 16	-
F total	All (*n* = 70)	18,803 ± 15,041	47 ± 24	51
WF	FITC (*n* = 20)	26,350 ± 15,525	62 ± 20	-
-	PE (*n* = 20)	12,586 ± 11,454	59 ± 18	-
-	APC (*n* = 20)	8470 ± 9490	57 ± 17	-
-	AF700 (*n* = 10)	20,911 ± 7871	43 ± 13	-
WF total	All (*n* = 70)	16,532 ± 13,764	57 ± 18	32

## Data Availability

Not applicable.

## Supplementary Materials

The following are available online at https://www.mdpi.com/2227-9059/9/2/206/s1, Figure S1: EV-size gate, Figure S2: Gating strategy, Figure S3: Scatterplots of PBS labeled with P2 specific, Figure S4: PBS scatterplots, Table S1: *p*-values for comparison of treatments, Figure S5: P1 specific labels, Figure S6: P1 isotype labels, Figure S7: P2 specific labels, Figure S8: P2 isotype labels, Table S2 Reduction (panels), Table S3: figure explanation for Figure 5, Figure S9: Scatterplot representation for the study presented in Figure 5, Table S4: reduction of individual labels for all treatments, Table S5: Points to consider when designing a label-panel.
