# Supplementary material for: Zoom in on Antibody Aggregates: A Potential Pitfall in the Search of Rare EV Populations
Source: Biomedicines. 2021 Feb 18;9(2):206. doi: 10.3390/biomedicines9020206 (PMC7923005; doi:10.3390/biomedicines9020206)
Supplement: Supplementary file 1 [file biomedicines-09-00206-s001.zip › Supplementary for publication/Figure S1.pdf]

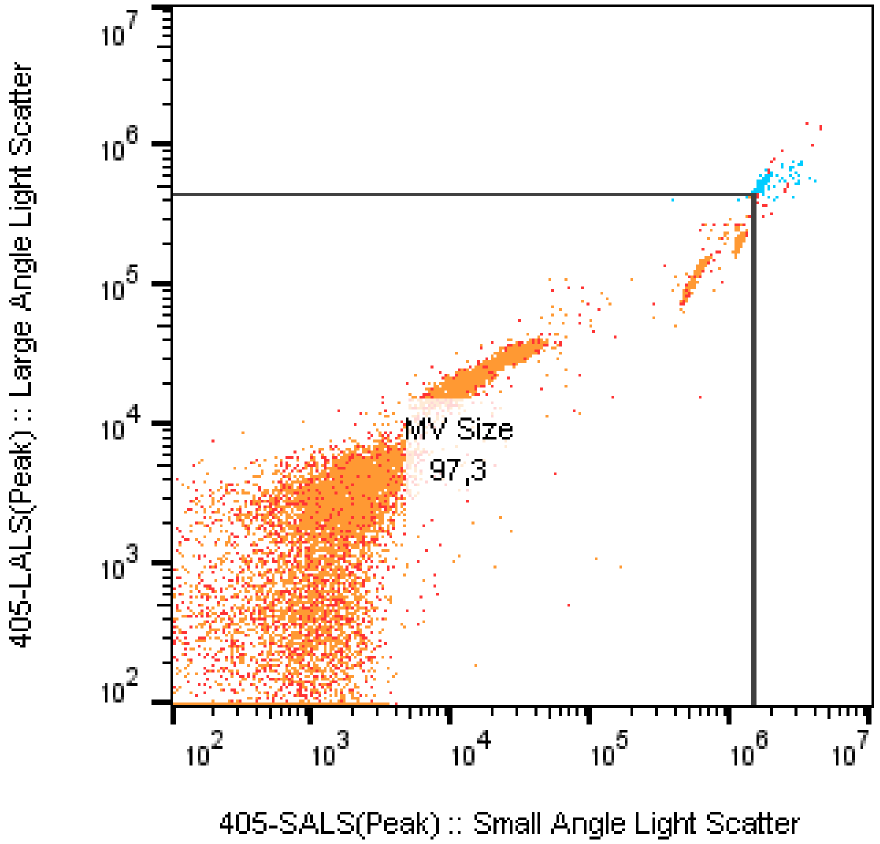

	Sample Name	Subset Name	Count
	20180626_QA_ApogeeMix_01_0.fcs	MV Size	103633
	20180626_QA_ApogeeMix_01_0.fcs	Si 1300	1001
	20180626_QA_ApogeeMix_01_0.fcs	Ungated	106515

Figure S1. MV-size gate was established by using ApogeeMix beads
