# Supplementary material for: Zoom in on Antibody Aggregates: A Potential Pitfall in the Search of Rare EV Populations
Source: Biomedicines. 2021 Feb 18;9(2):206. doi: 10.3390/biomedicines9020206 (PMC7923005; doi:10.3390/biomedicines9020206)
Supplement: Supplementary file 1 [file biomedicines-09-00206-s001.zip › Supplementary for publication/Figure S2_Gatingstrategy.pdf]

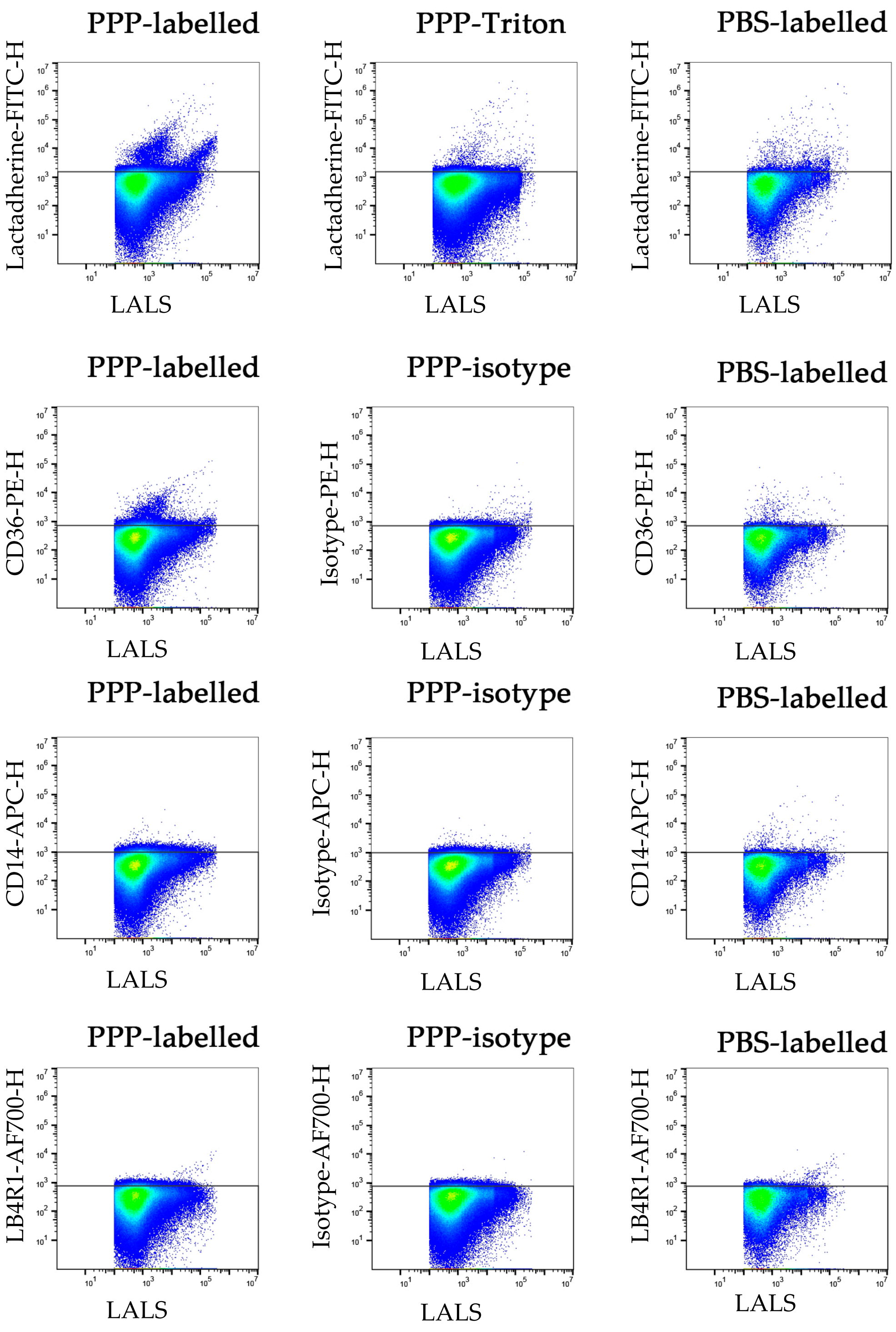

Figure S2 Gating strategy. All gates were set on PPP and transferred to PBS samples. 99percentile for lactadherine-FITC were set on the triton sample, and for other fluorophores on the isotype labelled sample.
