# Supplementary material for: Zoom in on Antibody Aggregates: A Potential Pitfall in the Search of Rare EV Populations
Source: Biomedicines. 2021 Feb 18;9(2):206. doi: 10.3390/biomedicines9020206 (PMC7923005; doi:10.3390/biomedicines9020206)
Supplement: Supplementary file 1 [file biomedicines-09-00206-s001.zip › Supplementary for publication/Figure S3_P2 scatterplots.pdf]

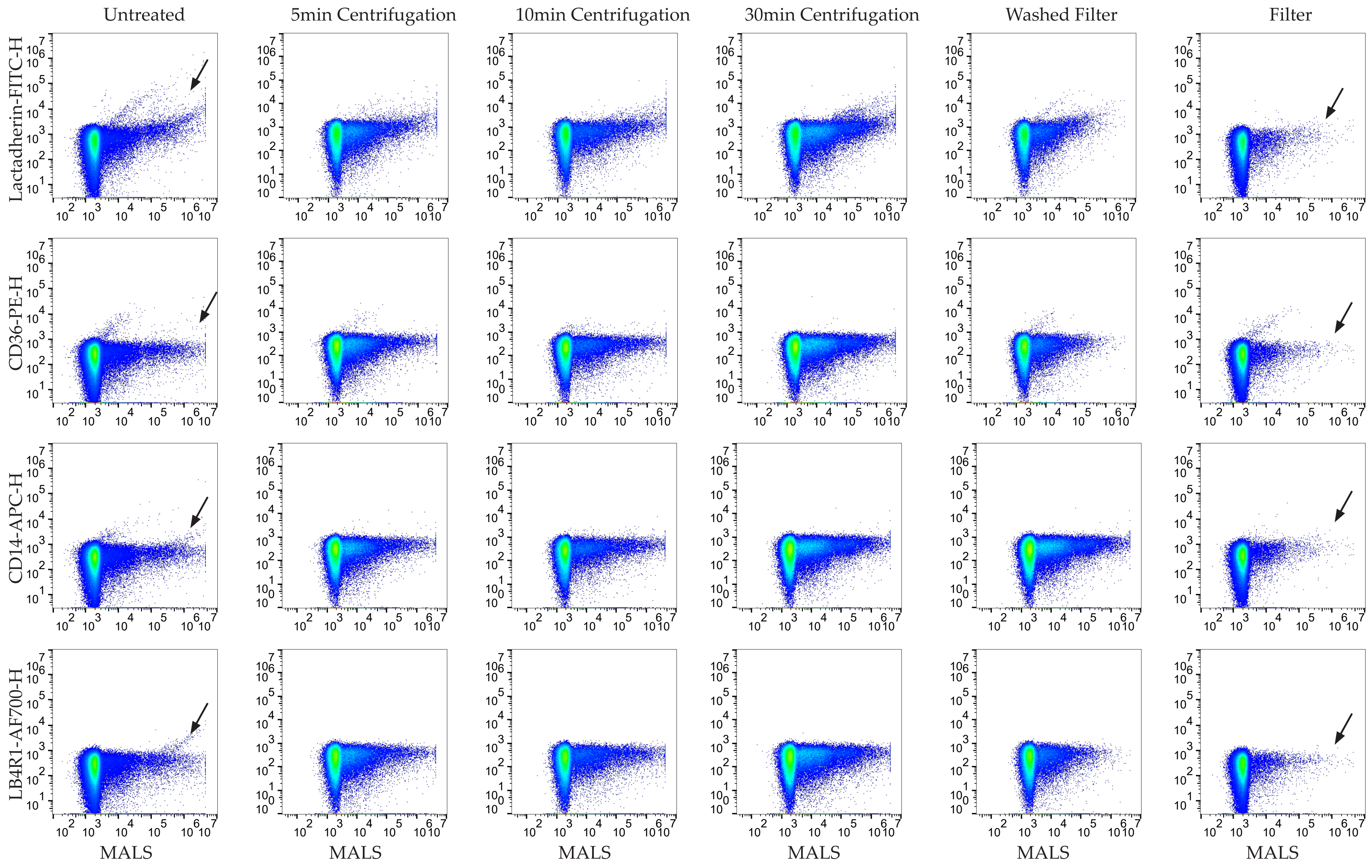

Figure S3 Representative scatterplots of PBS labelled with P2 specific label. Medium angle light scatter (MALS) versus fluorescence. Colum's represent untreated, centrifugated and filtered labels.
