# Supplementary material for: Zoom in on Antibody Aggregates: A Potential Pitfall in the Search of Rare EV Populations
Source: Biomedicines. 2021 Feb 18;9(2):206. doi: 10.3390/biomedicines9020206 (PMC7923005; doi:10.3390/biomedicines9020206)
Supplement: Supplementary file 1 [file biomedicines-09-00206-s001.zip › Supplementary for publication/Figure S7_P2 specific.pdf]

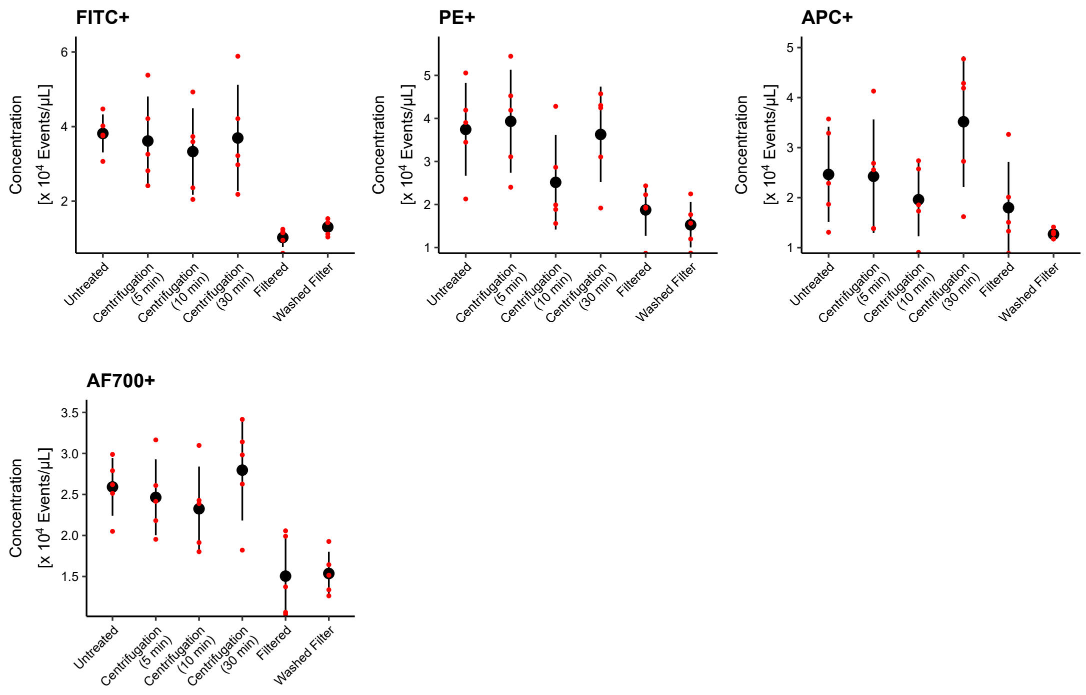


**Figure S7** P2, specific. Concentrations of FITC, PE, APC and AF700 aggregates in PBS labelled with P2 specific labels. Each bar corresponds to pre-treatment as indicated at the x-axis.
