# Supplementary material for: Zoom in on Antibody Aggregates: A Potential Pitfall in the Search of Rare EV Populations
Source: Biomedicines. 2021 Feb 18;9(2):206. doi: 10.3390/biomedicines9020206 (PMC7923005; doi:10.3390/biomedicines9020206)
Supplement: Supplementary file 1 [file biomedicines-09-00206-s001.zip › Supplementary for publication/Figure S9_multiple EV aggregates.pdf]

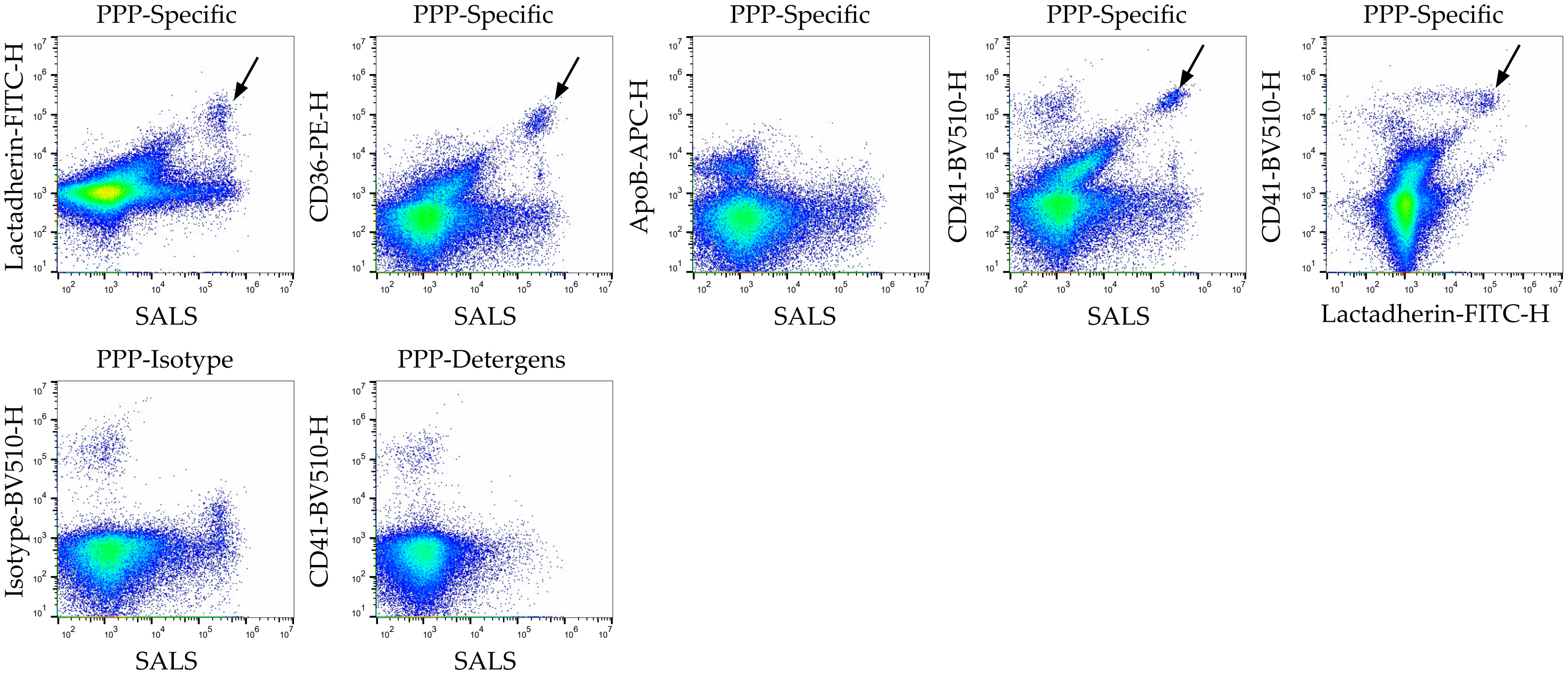

Figure S9 SALS vs. fluorescence or lactadherin-FITC vs. CD41-BV510 scatterplots of PPP labelled with either lactadherin-FITC, CD41-BV510, CD36-PE and ApoB-APC or corresponding isotypes and lactaherin-FITC. PPP-detergens is specific labelled PPP treated with Triton-X100. Arrows indicates aggregates + multiple EVs.
