# Supplementary material for: Zoom in on Antibody Aggregates: A Potential Pitfall in the Search of Rare EV Populations
Source: Biomedicines. 2021 Feb 18;9(2):206. doi: 10.3390/biomedicines9020206 (PMC7923005; doi:10.3390/biomedicines9020206)
Supplement: Supplementary file 1 [file biomedicines-09-00206-s001.zip › Supplementary for publication/Table S1.pdf]

Table S1. Statistical comparison of each treatment to any other treatment, for P1-specific or isotype and P2 specific or isotype, both for each label alone (FITC, PE, APC and AF700) or combined (All labels) for P1-specific, P1-isotype, P2-specific or P2-isotype, and combined for all panels and all labels. Statistical significance was defined as $p<0.05$. (shaded) =p<0.05.

		FITC[1]					PE[1]					APC[1]					AF700[1]					All labels				
		C10	C30	C5	F	U	C10	C30	C5	F	U	C10	C30	C5	F	U	C10	C30	C5	F	U	C10	C30	C5	F	U
P1 specific	C30	0.69	NA	NA	NA	NA	0.69	NA	NA	NA	NA	0.69	NA	NA	NA	NA	NA	NA	NA	NA	NA	0.841	NA	NA	NA	NA
	C5	0.421	0.841	NA	NA	NA	0.421	0.421	NA	NA	NA	0.095	0.151	NA	NA	NA	NA	NA	NA	NA	NA	0.192	0.369	NA	NA	NA
	F	0.151	0.032	0.032	NA	NA	0.095	0.222	0.056	NA	NA	0.016	0.222	0.841	NA	NA	NA	NA	NA	NA	NA	0.192	0.052	0.005	NA	NA
	U	0.841	0.69	0.69	0.548	NA	0.31	0.222	1	0.016	NA	0.151	0.056	0.421	0.421	NA	NA	NA	NA	NA	NA	0.547	0.547	0.62	0.043	NA
	WF	0.151	0.056	0.056	1	0.31	0.095	0.151	0.016	0.548	0.032	0.151	0.69	0.008	0.008	0.151	NA	NA	NA	NA	NA	0.006	0.023	<0.001	0.201	0.012
P1 isotype	C30	0.151	NA	NA	NA	NA	0.032	NA	NA	NA	NA	0.032	NA	NA	NA	NA	NA	NA	NA	NA	NA	0.004	NA	NA	NA	NA
	C5	1	0.31	NA	NA	NA	0.69	0.056	NA	NA	NA	0.548	0.056	NA	NA	NA	NA	NA	NA	NA	NA	0.583	0.026	NA	NA	NA
	F	0.056	0.31	0.032	NA	NA	0.056	0.222	0.222	NA	NA	0.548	0.016	0.421	NA	NA	NA	NA	NA	NA	NA	0.017	0.547	0.056	NA	NA
	U	0.31	0.421	0.548	0.222	NA	0.841	0.151	0.841	0.421	NA	0.31	0.016	0.222	0.841	NA	NA	NA	NA	NA	NA	0.659	0.017	0.82	0.052	NA
	WF	0.032	0.056	0.008	1	0.151	0.032	1	0.095	0.151	0.095	0.548	0.31	0.421	0.151	0.151	NA	NA	NA	NA	NA	<0.001	0.478	<0.001	0.142	0.003
P2 specific	C30	0.841	NA	NA	NA	NA	0.151	NA	NA	NA	NA	0.151	NA	NA	NA	NA	0.222	NA	NA	NA	NA	0.017	NA	NA	NA	NA
	C5	0.69	1	NA	NA	NA	0.095	0.841	NA	NA	NA	0.841	0.095	NA	NA	NA	0.548	0.421	NA	NA	NA	0.148	0.209	NA	NA	NA
	F	0.008	0.008	0.008	NA	NA	0.69	0.056	0.016	NA	NA	0.69	0.056	0.421	NA	NA	0.095	0.032	0.032	NA	NA	<0.001	<0.001	<0.001	NA	NA
	U	0.31	0.69	0.69	0.008	NA	0.151	1	0.841	0.032	NA	0.421	0.222	1	0.31	NA	0.31	0.421	0.548	0.016	NA	0.104	0.289	0.923	<0.001	NA
	WF	0.008	0.008	0.008	0.222	0.008	0.151	0.016	0.008	0.421	0.016	0.151	0.008	0.032	0.222	0.032	0.032	0.016	0.008	1	0.008	<0.001	<0.001	<0.001	0.758	<0.001
P2 isotype	C30	0.421	NA	NA	NA	NA	0.151	NA	NA	NA	NA	0.222	NA	NA	NA	NA	0.548	NA	NA	NA	NA	0.068	NA	NA	NA	NA
	C5	0.151	0.095	NA	NA	NA	0.69	0.056	NA	NA	NA	0.69	0.31	NA	NA	NA	0.841	0.31	NA	NA	NA	0.923	0.016	NA	NA	NA
	F	0.008	0.008	0.008	NA	NA	0.032	0.056	0.008	NA	NA	0.222	0.548	0.222	NA	NA	0.095	0.222	0.008	NA	NA	<0.001	<0.001	<0.001	NA	NA
	U	1	0.69	0.222	0.008	NA	1	0.032	0.421	0.008	NA	0.548	0.032	0.421	0.032	NA	0.151	0.095	0.31	0.008	NA	0.441	0.002	0.407	<0.001	NA
	WF	0.008	0.008	0.008	0.841	0.008	0.016	0.016	0.008	0.69	0.008	0.016	0.008	0.016	0.056	0.008	0.032	0.056	0.008	0.548	0.008	<0.001	<0.001	<0.001	0.386	<0.001
All panels	C30	NA	NA	NA	NA	NA	NA	NA	NA	NA	NA	NA	NA	NA	NA	NA	NA	NA	NA	NA	NA	0.365	NA	NA	NA	NA
	C5	NA	NA	NA	NA	NA	NA	NA	NA	NA	NA	NA	NA	NA	NA	NA	NA	NA	NA	NA	NA	0.26	0.05	NA	NA	NA
	F	NA	NA	NA	NA	NA	NA	NA	NA	NA	NA	NA	NA	NA	NA	NA	NA	NA	NA	NA	NA	<0.001	<0.001	<0.001	NA	NA
	U	NA	NA	NA	NA	NA	NA	NA	NA	NA	NA	NA	NA	NA	NA	NA	NA	NA	NA	NA	NA	0.132	0.017	0.582	<0.001	NA
	WF	NA	NA	NA	NA	NA	NA	NA	NA	NA	NA	NA	NA	NA	NA	NA	NA	NA	NA	NA	NA	<0.001	<0.001	<0.001	0.05	<0.001

[1] Fluorophores of the respective panel. See details of labels in Table 1. C5, C10 and C30: 5, 10 and 30min centrifugation. F: filtered. WF: washed filter. (shaded) =p<0.05
