# Supplementary material for: Zoom in on Antibody Aggregates: A Potential Pitfall in the Search of Rare EV Populations
Source: Biomedicines. 2021 Feb 18;9(2):206. doi: 10.3390/biomedicines9020206 (PMC7923005; doi:10.3390/biomedicines9020206)
Supplement: Supplementary file 1 [file biomedicines-09-00206-s001.zip › Supplementary for publication/Table S2.pdf]

Table S2. Reduction (%) of mean event concentrations (evt/μl) of P1 and P2 in PBS treated with either 5, 10 or 30min centrifugation (C5, C10 or C30)or filtered (F) or washed filter (WF), compared to evt/μl in PBS labelled with untreated labels (Table 3)

Treatment	Panel (all fluorophores)	Mean conc.±SD (events/μl)	Reduction % (compared to U)±SD
C5	P1 specific (*n*=15)	31823±42899	7±40
	P1 isotype (*n*=15)	31922±46637	13±49
	P2 specific (*n*=20)	31110±11812	2±31
	P2 isotype (*n*=20)	85901±71794	-10±56
C5 total	All (n=70)	47091±53701	2±45
C10	P1 specific (*n*=15)	26140±36152	23±36
	P1 isotype (*n*=15)	34863±48642	2±50
	P2 specific (*n*=20)	25340±9858	19±27
	P2 isotype (*n*=20)	68399±40523	7±33
C10 total	All (*n*=70)	39845±39594	13±37
C30	P1 specific (*n*=15)	29274±40968	21±45
	P1 isotype (*n*=15)	17192±25533	56±30
	P2 specific (*n*=20)	34105±11232	-11±40
	P2 isotype (*n*=20)	58088±32226	21±23
C30	All (*n*=70)	36298±31979	19±42
F	P1 specific (*n*=15)	13648±18583	44±20
	P1 isotype (*n*=15)	11891±15854	42±27
	P2 specific (*n*=20)	15524±6584	48±27
	P2 isotype (*n*=20)	31133±10417	53±20
F total	All (*n*=70)	18803±15041	47±24
WF	P1 specific (*n*=15)	13459±19306	53±20
	P1 isotype (*n*=15)	9989±13941	60±24
	P2 specific (*n*=20)	14114±3180	54±13
	P2 isotype (*n*=20)	26162±10724	61±17
WF total	All (*n*=70)	16532±13764	57±18
