# Supplementary material for: Zoom in on Antibody Aggregates: A Potential Pitfall in the Search of Rare EV Populations
Source: Biomedicines. 2021 Feb 18;9(2):206. doi: 10.3390/biomedicines9020206 (PMC7923005; doi:10.3390/biomedicines9020206)
Supplement: Supplementary file 1 [file biomedicines-09-00206-s001.zip › Supplementary for publication/Table S3.pdf]

Table S3: Schematic representations of the shapes and types of the different labels used in the study represented in Figure 5.

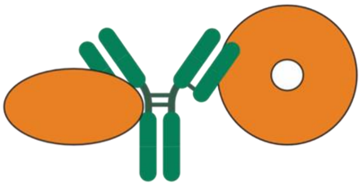<br>IgG-PE	Large and heavy biological fluorophores as phycobilliproteins that is globular proteins isolated from red algae.<br>IgG: 150kDa<br>APC: 105kDa<br>PE: 223kDa
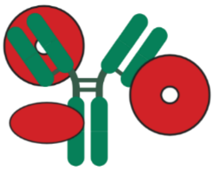<br>IgG-APC	
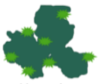<br>Lactadherine-FITC	Smaller and lighter organic dyes.<br>FITC: 0,590kDa<br>AF700: 1,4kDa Lactadherin: 53-66kDa
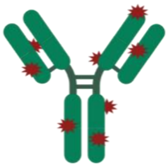<br>IgG-AF700	
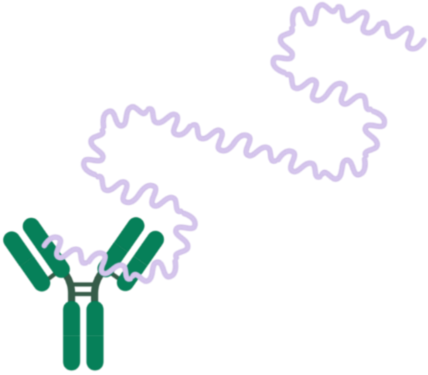<br>IgG-BV510	Organic polymers consisting of aromatic units and side chain modifications<br>BV510: 77kDa
