# Supplementary material for: Zoom in on Antibody Aggregates: A Potential Pitfall in the Search of Rare EV Populations
Source: Biomedicines. 2021 Feb 18;9(2):206. doi: 10.3390/biomedicines9020206 (PMC7923005; doi:10.3390/biomedicines9020206)
Supplement: Supplementary file 1 [file biomedicines-09-00206-s001.zip › Supplementary for publication/Table S5.pdf]

Table S6 Points to consider when designing a label-panel and protocol for a study of EVs by flow cytometry.

Operator and workflow protocol	Label and master mix of labels	Sample type	Data acquisition
F method: consider type of filter: size of pores, hydrophobic/hydrophilic filter	Ab-sequence, and fluorophore, is it prone for aggregation?	Solvent properties (PBS vs. protein/lipid containing organic solvent as plasma)	Fluorescence threshold
Temperature and incubation time	Solvent properties: pH<br>-protein concentration<br>-ionic strength<br>-hydrophobic surface -area in solvent	Rare or abundant EV-populations, is the number of aggregates significant compared to EVs?	Aggregates with one type of fluorophore or multiple?
Vortexing versus mixing by cautious pipetting	Interaction of labels when combining stocks in master mix		Aggregate/EV complexes.
Sequential labelling versus labelling with a master mix of labels.	Lowest possible label concentration in sample.		Appropriate controls as labelled PBS and detergent treated samples
