# Supplementary figures and images for: Zoom in on Antibody Aggregates: A Potential Pitfall in the Search of Rare EV Populations
Source: Biomedicines. 2021 Feb 18;9(2):206. doi: 10.3390/biomedicines9020206 (PMC7923005; doi:10.3390/biomedicines9020206)

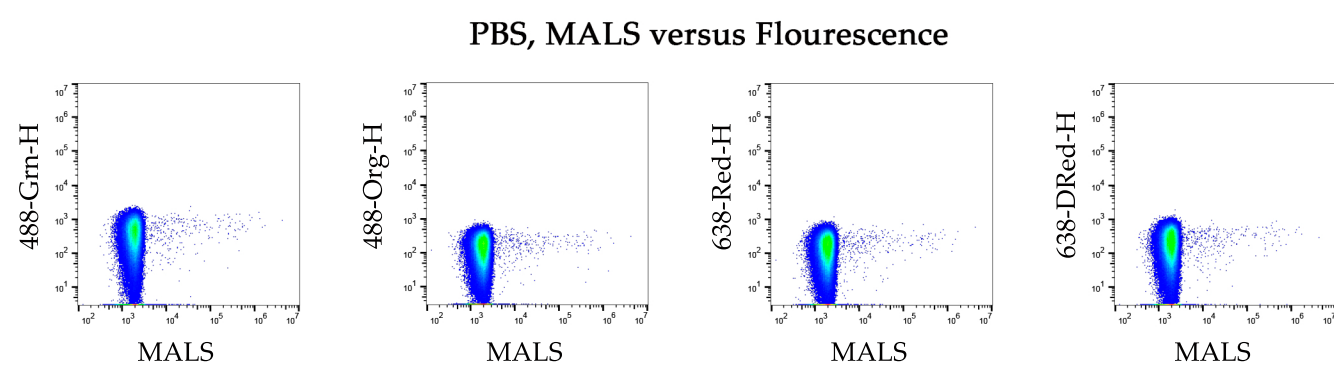

**Figure S4** Scatterplots of PBS only, Medium angle light scatter versus fluorescence.

Supplement: Supplementary file 1 [file biomedicines-09-00206-s001.zip › Supplementary for publication/Figure S4_PBS.pdf]
